# Early Auditory Stimulation, Not Device Type: Comparable Cortical Maturation in Children Using Cochlear Implants or Hearing Aids

**DOI:** 10.3390/children13050657

**Published:** 2026-05-08

**Authors:** Koray Tumuklu, Behcet Gunsoy

**Affiliations:** 1 Department of Otolaryngology, Private Clinic, 27310 Gaziantep, Turkey; 2 Department of Otorhinolaryngology, Faculty of Medicine, Gaziantep University, 27310 Gaziantep, Turkey; bgunsoy@gantep.edu.tr

**Keywords:** cortical auditory evoked potentials, P1 latency, cochlear implant, hearing aids, cortical maturation, pediatric hearing loss

## Abstract

**Highlights:**

**What are the main findings?**

• P1 latency did not show a statistically significant correlation with age at device fitting, indicating considerable inter-individual variability.
• Children with appropriate auditory support achieved comparable cortical auditory responses regardless of fitting age within the studied range.

**What are the implications of the main findings?**

• Age at device fitting alone may not be a sufficient predictor of cortical auditory maturation, highlighting the need to consider additional clinical and environmental factors.
• Early and effective auditory rehabilitation remains critical, but individualized follow-up strategies are essential to optimize neurodevelopmental outcomes.

**Abstract:**

**Introduction:** The present study aimed to compare cortical auditory maturation, as reflected by P1 latency of cortical auditory evoked potentials (CAEPs), in children with congenital severe-to-profound sensorineural hearing loss rehabilitated with unilateral cochlear implants (CIs) or bilateral conventional hearing aids (HAs). **Materials and Methods:** Eighty-five children with congenital severe-to-profound sensorineural hearing loss were included in this retrospective comparative study. Participants were divided into two groups: unilateral CI users (n = 42) and bilateral HA users (n = 43). All children were fitted with their devices before 48 months of age and achieved aided free-field thresholds between 30 and 50 dB HL. CAEPs were recorded using the Fonix^®^ HEARLab System with speech stimuli (/m/, /g/, /t/) presented at 55 dB SPL. P1 latency values were measured and compared between groups using independent samples *t*-tests. Correlation analyses were performed to assess the relationship between duration of device use and P1 latency. **Results:** Eighty-five children were included (CI: n = 42; HA: n = 43). Mean P1 latency values did not differ significantly between groups for the /m/ stimulus (126.4 ± 29.13 ms vs. 126.4 ± 29.28 ms, *p* = 1.00), /g/ stimulus (106.5 ± 26.46 ms vs. 110.1 ± 29.49 ms, *p* = 0.55), or /t/ stimulus (114.7 ± 22.93 ms vs. 118.5 ± 27.19 ms, *p* = 0.48). Age at device fitting was comparable between groups (27.95 ± 9.10 vs. 26.88 ± 14.15 months, *p* = 0.68). The duration of device use was significantly longer in the HA group (48.02 ± 28.39 months) compared to the CI group (26.00 ± 15.92 months) (*p* < 0.001). Correlation analysis revealed no significant association between duration of device use and P1 latency for any stimulus (/m/: *p* = 0.28; /g/: *p* = 0.17; /t/: *p* = 0.09). **Conclusions:** When devices were fitted before 48 months of age and aided thresholds were optimized, unilateral cochlear implantation and bilateral conventional hearing aids showed comparable P1 latency values as an index of cortical auditory maturation. These findings suggest that early and adequate auditory stimulation may play an important role in supporting cortical auditory development in children with congenital hearing loss, although results should be interpreted within the context of individualized clinical management.

## 1. Introduction

Congenital sensorineural hearing loss (SNHL) profoundly affects the maturation of the central auditory system by depriving the auditory cortex of essential acoustic stimulation during critical periods of neurodevelopment [1,2]. Auditory input is not merely sensory information; it acts as a biological driver of synaptic organization, dendritic arborization, and thalamocortical connectivity within the auditory pathways [3,4,5]. In the absence of appropriate stimulation, synaptic density is reduced, cortical layers reorganize abnormally, and cross-modal plasticity may occur, potentially compromising later auditory and language outcomes [6,7,8].

Neuroplasticity within the auditory system is highly time-dependent. Evidence from both animal models and human electrophysiological studies has demonstrated the existence of a sensitive period during which adequate auditory stimulation can induce near-normal cortical maturation [9,10,11]. Cochlear implantation (CI) and appropriately fitted hearing aids (HA) provide such stimulation by restoring access to acoustic input, thereby facilitating reactivation of thalamocortical projections and promoting cortical reorganization [12,13,14,15]. However, the degree to which different amplification modalities support cortical maturation remains a subject of ongoing investigation.

Cortical auditory evoked potentials (CAEPs) have emerged as reliable and objective neurophysiological markers of auditory cortical development. Among CAEP components, the P1 cortical response has gained particular clinical significance [16,17,18,19]. The P1 wave is a prominent positive peak generated primarily by auditory thalamic and cortical sources and reflects synaptic transmission along central auditory pathways [20,21]. Importantly, P1 latency decreases systematically with age, paralleling the maturation of cortical circuitry [20,21]. In normal-hearing infants, mean P1 latency is approximately 300 ms at birth, decreases to around 125 ms by 3 years of age, 85–95 ms by 5–6 years, and stabilizes near 60 ms in adulthood [15,22,23,24].

Because of this predictable developmental trajectory, P1 latency is widely accepted as an objective biomarker of central auditory maturation in children with hearing impairment [25,26,27]. Shortening of P1 latency following amplification indicates restoration of auditory input sufficient to support cortical development. Conversely, prolonged or abnormal P1 morphology may suggest insufficient stimulation or delayed cortical maturation [28,29,30].

While numerous studies have compared cochlear implants and hearing aids in terms of speech perception, language acquisition, and functional auditory outcomes—with mixed and sometimes conflicting results—objective neurophysiological comparisons using CAEP measures remain relatively limited. Behavioral measures can be influenced by cognitive, environmental, and rehabilitative variables, whereas CAEPs provide a direct electrophysiological assessment of cortical auditory processing independent of subjective cooperation.

Furthermore, unilateral cochlear implantation and bilateral conventional hearing aid use represent distinct modes of auditory rehabilitation. Cochlear implants deliver electrical stimulation directly to the auditory nerve, bypassing damaged hair cells, whereas hearing aids amplify acoustic signals that depend on residual cochlear function. Whether these differing mechanisms result in measurable differences in cortical maturation—particularly when intervention occurs early in life—remains clinically relevant.

Given the established relationship between auditory stimulation and cortical plasticity, and considering the importance of early intervention in congenital SNHL, objective comparison of cortical maturation between children using unilateral cochlear implants and those using bilateral hearing aids is warranted.

Therefore, the aim of the present study was to compare P1 latencies obtained through cortical auditory evoked potentials in children with congenital severe-to-profound sensorineural hearing loss who were rehabilitated either with unilateral cochlear implants or bilateral conventional hearing aids.

## 2. Materials and Methods

### 2.1. Study Design and Participants

This retrospective comparative study was conducted at the Department of Otolaryngology and Audiology following approval from the Gaziantep University Non-Interventional Clinical Research Ethics Committee (Date: 5 November 2025; Decision No: 2025/384). The study was performed in accordance with the principles of the Declaration of Helsinki. Written informed consent had been obtained from the parents or legal guardians of all participants prior to audiological and electrophysiological evaluations.

Children diagnosed with bilateral congenital severe-to-profound sensorineural hearing loss (SNHL) who had been rehabilitated either with unilateral cochlear implants (CI) or bilateral conventional hearing aids (HA) were included in the study. Medical and audiological records of all eligible participants were systematically reviewed. The extracted data included age at the time of electrophysiological assessment, sex, age at device fitting, duration of device use, unaided pure-tone hearing thresholds, aided free-field hearing thresholds, and P1 latency values obtained from cortical auditory evoked potential (CAEP) recordings.

### 2.2. Inclusion and Exclusion Criteria

Children were included in the study if they had congenital severe-to-profound sensorineural hearing loss, defined as unaided pure-tone thresholds greater than 70 dB HL, and achieved aided free-field thresholds between 30 and 50 dB HL. All participants had been fitted with either a unilateral cochlear implant or bilateral conventional hearing aids before 48 months of age and had a minimum device experience of six months. In addition, all children were regularly enrolled in aural–oral rehabilitation programs following device fitting.

**Figure 1:**
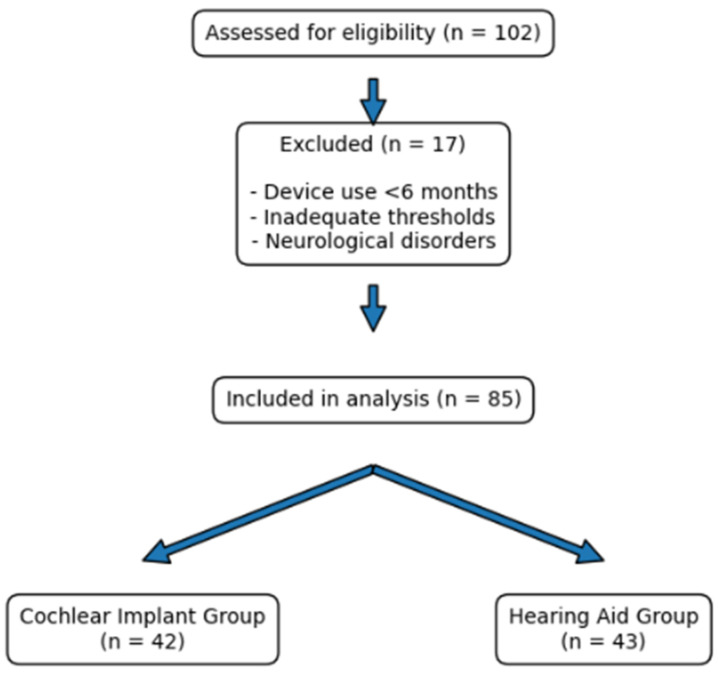
Flowchart of the study.

Participants with unaided thresholds better than 70 dB HL, corresponding to mild-to-moderate or moderate hearing loss rather than severe-to-profound hearing loss, were excluded. Children with neurological disorders, conditions affecting central auditory pathways, or additional cognitive or developmental impairments that could influence cortical auditory responses were also excluded. A total of 85 children met the eligibility criteria and were included in the final analysis (Figure 1).

Demographic, clinical, and audiometric characteristics, including unaided hearing thresholds, are presented in Table 1. A total of 85 children who met the inclusion criteria were included in the final analysis. Of these, 42 children were fitted with unilateral cochlear implants (CI group) and 43 with bilateral hearing aids (HA group). The CI group included 22 females and 20 males, while the HA group included 21 females and 22 males. The mean age at assessment was 57.52 ± 18.99 months in the CI group and 74.77 ± 30.86 months in the HA group. Unaided pure-tone average (PTA) values were significantly higher in the CI group than in the HA group (*p* < 0.001) (Table 1).

### 2.3. Audiological Assessment, Cortical Auditory Evoked Potentials (CAEPs) and Recording Procedure

Unaided and aided hearing thresholds were calculated at four frequencies (500 Hz, 1000 Hz, 2000 Hz, and 4000 Hz) using free-field audiometry. Unaided hearing thresholds were also analyzed as continuous variables to better characterize the severity of hearing loss within each group. Threshold averages were obtained to confirm eligibility criteria. All hearing aid fittings and cochlear implant mappings were performed by experienced audiologists using standard pediatric fitting protocols. For hearing aid users, amplification targets were determined according to pediatric prescriptive fitting formulas. Cochlear implant users underwent routine mapping adjustments based on behavioral and objective measures.

Cortical auditory evoked potentials were recorded using the Fonix^®^ HEARLab System (Frye Electronics, Inc., Beaverton, OR, USA), which provides automated statistical detection of cortical responses [31,32]. Participants were tested while awake in a seated and relaxed position. Testing was performed in a sound-treated room, with the loudspeaker positioned at 0° azimuth directly in front of the participant, approximately 1 m from the child’s head. Cortical auditory evoked potentials were recorded using surface electrodes placed at the vertex (Cz) as the active electrode and on the forehead as the reference electrode. Electrode impedance was maintained within clinically acceptable limits throughout the recording session. The /m/ stimulus was considered low-frequency dominant because its acoustic energy is mainly related to voicing and nasal resonance in the lower-frequency region, particularly around the first formant region. The /g/ stimulus was considered to represent mid-frequency speech information because its spectral cues are concentrated predominantly in the mid-frequency range. The /t/ stimulus was considered high-frequency dominant due to its voiceless stop characteristics and higher-frequency spectral burst components, including energy in the upper speech frequency region. Therefore, these three stimuli were used to sample cortical responses across low-, mid-, and high-frequency regions of the speech spectrum. These stimuli were selected because they reflect key components of conversational speech across low, mid, and high frequency regions of the auditory spectrum [32,33]. The HEARLab system automatically performed statistical analysis for each stimulus and calculated a *p*-value to determine the presence of a cortical response. A response was considered present when *p* < 0.05.

The first robust positive waveform occurring between 50 and 300 ms after stimulus onset was identified as the P1 cortical response [22,34]. P1 latency was defined as the time interval, measured in milliseconds, from stimulus onset to the peak amplitude of the P1 waveform. Latency measurements were obtained separately for each speech stimulus (/m/, /g/, and /t/). P1 peak identification was performed based on standard criteria, defined as the first prominent positive waveform occurring between 50 and 300 ms following stimulus onset. All waveforms were visually inspected by an experienced audiologist to confirm automated detection results. In cases of ambiguous or low-amplitude responses, recordings were re-evaluated to ensure accurate peak identification. All participants included in the analysis demonstrated clearly identifiable P1 responses for the evaluated stimuli.

### 2.4. Statistical Analysis

All statistical analyses were performed using SPSS software v27 (IBM Corp., Armonk, NY, USA). Continuous variables were expressed as mean ± standard deviation (SD), while categorical variables were presented as frequencies and percentages. Normality of continuous variables was assessed prior to parametric testing. Between-group comparisons (cochlear implant vs. hearing aid groups) were conducted using independent samples Student’s *t*-test for normally distributed continuous variables, including P1 latencies, age at device fitting, and duration of device use. Pearson correlation analysis was performed to evaluate the relationship between duration of device use and P1 latency. In addition, analysis of covariance (ANCOVA) was performed to compare P1 latency values between the cochlear implant (CI) and hearing aid (HA) groups while controlling for potential confounding variables. Age at assessment and duration of device use were included as covariates. Adjusted mean values were calculated, and between-group differences were evaluated after controlling for these variables. In addition, 95% confidence intervals (CIs) were calculated for all primary comparisons to provide an estimate of the precision of the observed effects. A *p*-value < 0.05 was considered statistically significant.

## 3. Results

**Figure 2:**
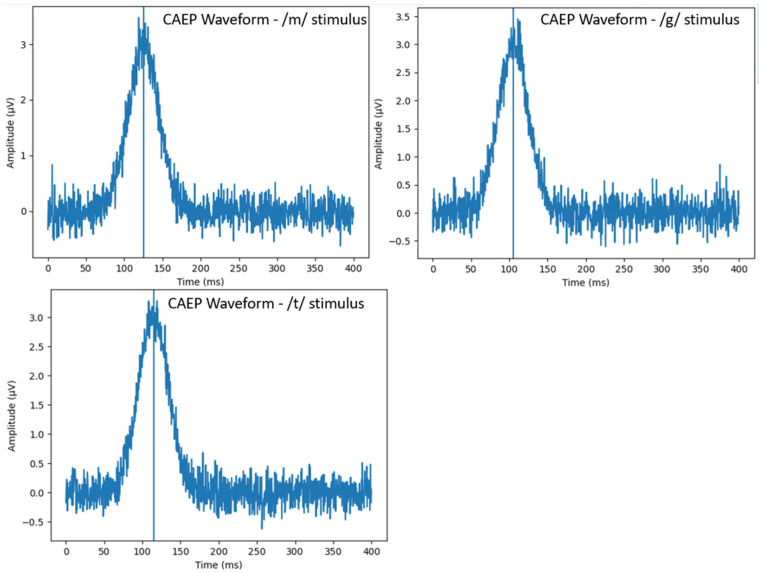
Representative cortical auditory evoked potential (CAEP) waveforms demonstrating P1 identification for /m/, /g/, and /t/ stimuli. The recordings are obtained from a single representative participant and illustrate typical waveform morphology observed across the study groups.

Mean P1 Latencies and Latency Ranges for Speech Stimuli (/m/, /g/, /t/) in CI and HA Groups were shown in Table 2. Mean P1 latencies for the /m/ stimulus were 126.4 ± 29.13 ms in the CI group and 126.4 ± 29.28 ms in the HA group. For the /g/ stimulus, mean P1 latencies were 106.5 ± 26.46 ms in the CI group and 110.1 ± 29.49 ms in the HA group. For the /t/ stimulus, mean P1 latencies were 114.7 ± 22.93 ms in the CI group and 118.5 ± 27.19 ms in the HA group (Table 2, Figure 2).

In addition to between-group comparisons, within-group analyses were performed to evaluate differences in P1 latency across the three speech stimuli (/m/, /g/, /t/). Repeated measures analysis demonstrated that P1 latency values varied across stimuli, with relatively shorter latencies observed for mid-frequency (/g/) stimuli compared to low-frequency (/m/) stimuli, although these differences did not reach statistical significance (*p* > 0.05). A similar pattern was observed in both CI and HA groups.

Independent samples *t*-test results are presented in Table 3. There was no significant difference in age at device fitting between the groups (*p* = 0.68). Duration of device use was significantly longer in the HA group (*p* < 0.001). No statistically significant between-group differences were observed in P1 latency values for any of the speech stimuli (/m/, /g/, /t/) (all *p* > 0.05) (Table 3).

**Figure 3:**
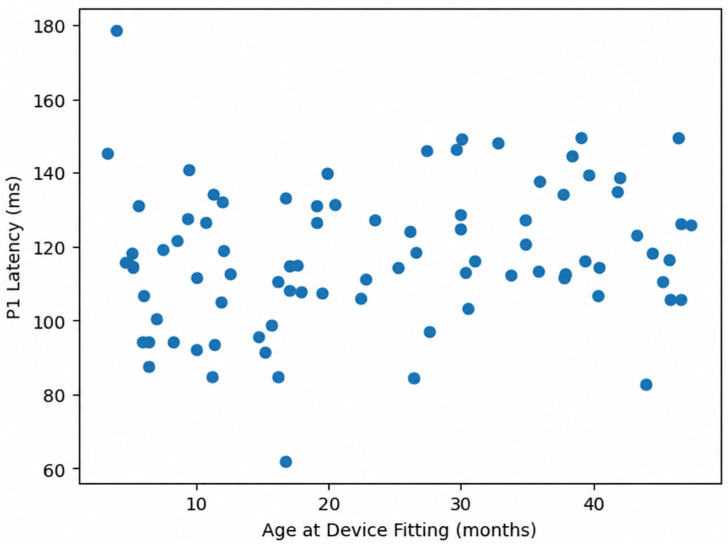
Scatter plot illustrating the relationship between age at device fitting and P1 latency. No regression line is shown, as the correlation was not statistically significant.

Correlation analysis between duration of device use and P1 latency values is presented in Table 4. Although weak negative trends were observed between duration of device use and P1 latency for all speech stimuli, these associations did not reach statistical significance (/m/: r = −0.12, *p* = 0.28; /g/: r = −0.15, *p* = 0.17; /t/: r = −0.18, *p* = 0.09) (Table 4, Figure 3).

To further account for potential confounding effects, an analysis of covariance (ANCOVA) was performed to compare P1 latency values between groups while controlling for age at assessment and duration of device use. After adjustment, no statistically significant differences were observed between the CI and HA groups for any of the speech stimuli (/m/, /g/, /t/) (all *p* > 0.05) (Table 5).

## 4. Discussion

The present study aimed to compare cortical auditory maturation, as reflected by P1 latency of cortical auditory evoked potentials (CAEPs), in children with congenital severe-to-profound sensorineural hearing loss rehabilitated with unilateral cochlear implants (CIs) or bilateral conventional hearing aids (HAs). The principal finding of this study was that no statistically significant differences were observed between CI and HA users in P1 latency values for low-, mid-, and high-frequency speech stimuli when devices were fitted before 48 months of age and aided thresholds were maintained between 30 and 50 dB HL. Another important consideration is the difference in auditory input configuration between the groups. In the present study, children in the hearing aid group received bilateral acoustic stimulation, whereas children in the cochlear implant group received unilateral electrical stimulation. Differences in binaural versus monaural input may influence cortical organization, including interhemispheric processing and auditory pathway development. Although our results did not demonstrate significant differences in P1 latency, the potential impact of binaural auditory input on higher-level auditory processing cannot be excluded and should be considered when interpreting the findings.

Auditory cortical development is highly dependent on early sensory input. Kral et al. demonstrated that congenital auditory deprivation leads to synaptic and laminar reorganization within the auditory cortex, particularly during critical developmental windows [2,6]. Chang et al. reported that proper thalamocortical connectivity requires timely acoustic stimulation for normal circuit refinement [4]. In the absence of adequate input, cross-modal reorganization may occur, potentially limiting later auditory outcomes [6,29]. Our findings suggested that when early and sufficient amplification is provided—either electrically via CI or acoustically via bilateral HA—cortical maturation, as indexed by P1 latency, progresses in a comparable manner. This aligns with Sharma et al., who established P1 latency as a reliable biomarker of central auditory development and demonstrated age-appropriate cortical responses following early cochlear implantation [24]. An important observation in the present study is that the mean P1 latency values in both groups were comparable to those reported in normal-hearing children of approximately 3 years of age, despite the mean chronological age of our cohort being closer to 5 years. This finding may suggest a degree of delayed cortical maturation, even in the presence of early and adequate auditory stimulation. Although amplification was provided within the sensitive period, cortical development may not fully reach age-appropriate levels in all children with congenital hearing loss. This discrepancy highlights the importance of considering both chronological age and electrophysiological maturation when interpreting CAEP findings.

HabibAllah et al. reported that CAEP components recorded directly through cochlear implants were associated with post-implantation speech and auditory outcomes, supporting the utility of cortical responses as objective markers of rehabilitation success [16,17]. Roslle et al. found that children with cochlear implants exhibited cortical maturation patterns approaching those of normal-hearing peers, particularly when implantation occurred within the sensitive period [18]. Rather than indicating superiority of one intervention over another, these findings emphasize the importance of timely and adequate access to auditory input. In the present study, no statistically significant differences were observed between unilateral cochlear implant users and bilateral hearing aid users in P1 latency values. This suggests that, when aided thresholds are appropriately optimized and intervention occurs early, cortical auditory maturation may be more strongly influenced by effective auditory stimulation than by device type alone. These results should be interpreted within a clinical framework in which device selection is guided by individual hearing levels, auditory characteristics, and the goal of achieving sufficient access to speech across the frequency spectrum.

Távora-Vieira et al. also demonstrated that CAEP responses in cochlear implant users reflect effective cortical encoding of speech stimuli across frequency bands [32]. In our study, similar latency values across /m/, /g/, and /t/ stimuli in both groups support the notion that adequate amplification—regardless of modality—can provide sufficient spectral information to support cortical development.

Kaplan-Neeman et al. investigated auditory cortical plasticity in children with unilateral hearing loss, where one ear had normal acoustic input while the other was rehabilitated [8]. Therefore, the population studied differs substantially from the present cohort, which consisted of children with bilateral congenital hearing loss. This distinction should be considered when interpreting comparisons, as cortical organization patterns may differ between unilateral and bilateral auditory deprivation. Lamminmäki et al. further emphasized that auditory cortex maturation in children with hearing loss is strongly associated with consistent device use and early intervention [33]. Our results extend previous findings by showing that bilateral hearing aid users with severe-to-profound hearing loss may achieve P1 latency values comparable to those of unilateral cochlear implant users when aided thresholds are adequately controlled. This finding highlights the importance of sufficient and timely auditory input in supporting cortical maturation.

In clinical practice, the selection between cochlear implants and hearing aids is based on individualized audiological assessment, with the primary goal of achieving adequate access to speech across the frequency spectrum. In cases where acoustic amplification cannot provide sufficient access—particularly in profound high-frequency hearing loss—cochlear implantation may be required. Conversely, when residual hearing allows effective amplification, hearing aids may provide appropriate auditory access. Therefore, cortical auditory maturation appears to be primarily influenced by the effectiveness of auditory stimulation rather than the specific device used, particularly under conditions of optimized aided thresholds and early intervention.

Although the duration of device use was significantly longer in the HA group, correlation analysis revealed only weak and non-significant associations between duration of use and P1 latency. Importantly, the absence of significant differences between groups persisted after adjusting for age at assessment and duration of device use in the ANCOVA analysis. This strengthens the validity of the findings by demonstrating that the observed similarities in P1 latency are unlikely to be solely explained by these confounding variables. Hajimohammadi et al., in their systematic review, suggested that CAEP changes may reflect auditory training and rehabilitation effects over time; however, maturation may plateau once sufficient cortical reorganization has occurred [20]. The absence of a strong correlation in our study may indicate that early intervention timing plays a more decisive role than total duration of use once a minimal threshold of cortical stimulation has been achieved. This interpretation is consistent with the sensitive period hypothesis proposed by Sharma et al. [24].

Cross-modal reorganization remains a critical issue in children with hearing loss. Hennesy et al. demonstrated that visual and somatosensory cross-modal plasticity in cochlear-implanted children may influence speech perception outcomes [27]. Simon et al. emphasized that delayed auditory input increases the likelihood of cortical reallocation to other sensory modalities [29]. The comparable P1 latencies observed in our CI and HA groups suggested that early bilateral acoustic stimulation may be sufficient to limit maladaptive cross-modal reorganization, similarly to cochlear implantation within the sensitive period.

Our findings support the use of P1 latency as an objective biomarker for monitoring cortical auditory maturation in children undergoing auditory rehabilitation. Gabr et al. highlighted that auditory evoked potentials provide valuable objective data in evaluating cochlear implant outcomes [21]. Extending this approach to hearing aid users provides clinicians with a neurophysiological framework to assess adequacy of amplification beyond behavioral measures. Importantly, the results indicate that bilateral hearing aids may achieve cortical maturation comparable to unilateral cochlear implants when fitted early and optimized to achieve functional aided thresholds. This has implications for device selection decisions, particularly in borderline candidacy cases.

Unlike many previously published studies that primarily focused on behavioral outcomes such as speech perception and language development when comparing cochlear implant and hearing aid users, the present study provided an objective neurophysiological comparison using P1 latency of cortical auditory evoked potentials. While earlier reports often suggested advantages of cochlear implantation in functional auditory performance, few studies directly evaluated cortical maturation between unilateral CI and bilateral HA users under controlled aided threshold conditions. By restricting inclusion to children fitted before 48 months of age and achieving comparable aided thresholds (30–50 dB HL), our study minimized confounding effects related to delayed intervention or insufficient amplification. This design allowed a more focused evaluation of cortical auditory development itself, rather than downstream behavioral outcomes, thereby distinguishing our findings from much of the existing literature.

Future prospective longitudinal studies incorporating multimodal neurophysiological measures and functional language outcomes are warranted. Direct comparisons between unilateral and bilateral cochlear implantation and bilateral hearing aid use may further elucidate the role of binaural stimulation in cortical maturation.

This study has some limitations. First, its retrospective design may introduce selection bias. Second, although age at fitting was similar between groups, the difference in duration of device use could potentially influence cortical measures. Third, we evaluated only P1 latency. It is important to recognize that the P1 component primarily reflects the arrival of auditory signals to the auditory cortex and early-stage cortical processing, and does not provide direct information regarding higher-level auditory functions such as speech perception, language comprehension, or cognitive processing. Therefore, while P1 latency is a valuable biomarker of cortical auditory maturation, it represents only one aspect of auditory function. Additional cortical components (e.g., N1, P2) and behavioral outcome measures would provide a more comprehensive evaluation of auditory processing and functional performance. In addition, the possibility of a type II error should be considered, as the sample size, although moderate, may not have been sufficient to detect small differences between groups. Therefore, non-significant findings should be interpreted with caution. Finally, behavioral speech and language outcomes were not analyzed in parallel with electrophysiological findings.

## 5. Conclusions

In conclusion, P1 latency appears to be a useful objective marker of cortical auditory maturation in children with congenital severe-to-profound sensorineural hearing loss. In the present study, no statistically significant differences were observed in P1 latency values between children using unilateral cochlear implants and those using bilateral conventional hearing aids when devices were fitted before 48 months of age and aided thresholds were optimized. These findings suggest that early and adequate auditory stimulation may play a key role in supporting cortical auditory development. However, the results should be interpreted within the context of individualized clinical decision-making, in which device selection is based on patient-specific audiological characteristics and the goal of achieving sufficient access to speech across the frequency spectrum. Objective electrophysiological monitoring using CAEPs may provide valuable complementary information for evaluating auditory rehabilitation in pediatric populations. Further studies incorporating additional electrophysiological and behavioral measures are warranted to better understand functional auditory outcomes.

## Figures and Tables

**Table 1:** Demographic and Clinical Characteristics of the Study Groups.

Variable	Cochlear Implant (n = 42)	Hearing Aid (n = 43)	*p*-Value
Age at assessment (months), mean ± SD	57.52 ± 18.99	74.77 ± 30.86	>0.05
Female, n (%)	22 (52.4%)	21 (48.8%)	>0.05
Male, n (%)	20 (47.6%)	22 (51.2%)	
Age at device fitting (months), mean ± SD	27.95 ± 9.10	26.88 ± 14.15	>0.05
Duration of device use (months), mean ± SD	26.00 ± 15.92	48.02 ± 28.39	<0.05 *
Unaided PTA (dB HL), mean ± SD	94.6 ± 7.8	81.3 ± 6.5	*p* < 0.001
Unaided threshold (dB HL)	>70	>70	—
Aided free-field threshold (dB HL)	30–50	30–50	—

* Statistically significant difference (*p* < 0.05).

**Table 2:** Mean P1 Latencies and Latency Ranges for Speech Stimuli (/m/, /g/, /t/) in CI and HA Groups.

Stimulus	Group	Mean P1 Latency (ms) ± SD	Latency Range (ms)	*p*-Value
**/m/** (Low frequency)	CI (n = 42)	126.4 ± 29.13	73–271	>0.05
	HA (n = 43)	126.4 ± 29.28	73–237	
**/g/** (Mid frequency)	CI (n = 42)	106.5 ± 26.46	70–225	>0.05
	HA (n = 43)	110.1 ± 29.49	60–218	
**/t/** (High frequency)	CI (n = 42)	114.7 ± 22.93	88–238	>0.05
	HA (n = 43)	118.5 ± 27.19	60–173	

**Table 3:** Independent Samples t-Test Results for Between-Group Comparisons.

Variable	Mean Difference (CI–HA)	95% CI	t	df	*p*-Value	Cohen’s d
Age at device fitting	1.07	−4.10 to 6.24	0.41	83	0.68	0.09
Duration of device use	−22.02	−32.30 to −11.74	−4.30	83	<0.001	0.98
P1 latency–/m/	0.00	−12.30 to 12.30	0.00	83	1.00	0.00
P1 latency–/g/	−3.60	−15.80 to 8.60	−0.60	83	0.55	0.13
P1 latency–/t/	−3.80	−14.90 to 7.30	−0.71	83	0.48	0.15

CI: Cochlear implant; HA: Hearing aid; CI–HA: mean difference between groups.

**Table 4:** Correlation Analysis Between Duration of Device Use and P1 Latencies.

Variable	r	*p*-Value
Duration of device use vs. P1 latency (/m/)	−0.12	0.28
Duration of device use vs. P1 latency (/g/)	−0.15	0.17
Duration of device use vs. P1 latency (/t/)	−0.18	0.09

**Table 5:** ANCOVA Results for Adjusted P1 Latency Comparisons.

Stimulus	Adjusted Mean (CI) ms	Adjusted Mean (HA) ms	F-Value	*p*-Value
/m/	125.8	127.1	0.08	0.77
/g/	107.2	109.4	0.42	0.52
/t/	115.3	117.6	0.39	0.53

Covariates: age at assessment, duration of device use.

## Data Availability

The data supporting the findings of this study are available from the corresponding author upon reasonable request.

## Abbreviations

CAEP	Cortical Auditory Evoked Potential
CI	Cochlear Implant
HA	Hearing Aid
SNHL	Sensorineural Hearing Loss
P1	First Positive Cortical Auditory Component
SD	Standard Deviation
dB HL	Decibel Hearing Level
dB SPL	Decibel Sound Pressure Level
Cz	Vertex Electrode Position
